# Temporal Pattern and Spatial Distribution of Tuberculosis Prevalence Associated with Multimorbidity in Brazil

**DOI:** 10.1590/0037-8682-0625-2023

**Published:** 2024-07-29

**Authors:** Bruno Victor Barros Cabral, George Jó Bezerra Sousa, Luana Ibiapina Cordeiro, Thatiana Araújo Maranhão, Maria Lúcia Duarte Pereira

**Affiliations:** 1 Universidade Estadual do Ceará, Departamento de Enfermagem, Fortaleza, CE, Brasil.; 2 Secretaria de Saúde do Ceará, Fortaleza, CE, Brasil.; 3 Universidade Estadual do Piauí, Departamento de Enfermagem, Parnaíba, PI, Brasil.

**Keywords:** Tuberculosis, Multimorbidity, Spatial Analysis

## Abstract

**Background:**

Four main chronic conditions may be involved in the tuberculosis pathogenic process and/or clinical evolution: HIV/AIDS, diabetes mellitus, mental illness, and Chronic Obstructive Pulmonary Disease. This study aimed to determine the spatiotemporal pattern of tuberculosis (TB) associated with multimorbidity in Brazil.

**Methods:**

Ecological study use data from the Notifiable Diseases Information System (SINAN), collected from the electronic portal of the Department of Informatics of the SUS (DATASUS). These data included TB cases that were reported between 2007 and 2021 and were associated with two or more chronic clinical health conditions (multimorbidity).

**Results:**

A total of 7,795 cases of TB associated with multimorbidity were recorded, with an average growth trend of 4.6% per year (95% Confidence Interval (CI): 3.3-5.9; p<0.001) and higher growth in the first temporal segment (2007 to 2011) (8.9%; 95%CI: 4.2-13.9; p=0.002). The spatial analysis, after Bayesian smoothing, highlighted the main municipalities states of the study, namely: São Paulo (19.8%; n = 297), Porto Alegre (23.6%; n = 354), and Rio de Janeiro (44.8%; n = 672). The proportion of extremely poor (β=-0.002), the *bolsa família* program (β=0.002), the average per-person income (β=0.001), and the percentage of the population living in households with a density of more than 2 people per bedroom (β=0.001) were related to chronic health conditions.

**Conclusions:**

These findings will stimulate public action to manage this situation. However, as this is still a recent topic in the literature, we encourage the development of studies on the synergistic characteristics of TB and other clinical conditions.

## INTRODUCTION

Tuberculosis (TB) is a public health concern. In 2022 alone, 7,5 million new cases were reported worldwide, and in the same year, 1,13 million died from this disease^1^. This magnitude is associated with factors that favor its evolution, such as HIV/AIDS, malnutrition, smoking, and alcoholism. In addition, the literature points to other chronic conditions that may be involved in the pathogenic process and/or clinical evolution, such as decompensated *diabetes mellitus* (DM), mental illness, and Chronic Obstructive Pulmonary Disease (COPD)^2^ ^-^ ^5^.

In this context, these diseases acquire syndemic characteristics of TB. Syndemic conditions correspond to the study of concomitant conditions that share similar pathophysiology and are predisposed by biological factors in such a way as to affect people, not only through somatic symptoms. Thus, in light of this theory, the aggregation of these diseases into TB is circumscribed in a biological context and has a background of social and economic disparities that are thus exacerbated^6^ ^,^ ^7^.

In addition, the combination of TB and the presence of other diseases has been increasingly reported, a context that has recently been called TB-associated multimorbidity. The World Health Organization (WHO) defines "multimorbidity" as the existence of two or more chronic health conditions in the same individual. In the context of TB, multimorbidity leads to low percentages of treatment adherence, polypharmacy, and difficulty in integrating specialized health services for each condition^8^ ^,^ ^9^. It is estimated that 30% of adults in developed countries have multimorbidity. Regarding TB, Jarde et al^10^., in a review on the subject, showed that there is a high prevalence of the phenomenon in Latin America and Africa.

Thus, a significant number of TB cases associated with chronic diseases, such as HIV, DM, mental illness, and COPD, are currently considered a global public health problem that has only recently begun to be studied in its syndemic aspect^11^. Few studies have identified the characteristics and clusters of TB associated with multimorbidity, weakening our knowledge of the distribution of this phenomenon in Brazil. Therefore, ecological studies are essential to formulate actions and public policies that contribute to reducing and eliminating TB worldwide. This study aimed to determine the temporal pattern and spatial distribution of the prevalence of tuberculosis (TB) associated with multimorbidity in Brazil.

## METHODS

This is an ecological study with temporal and spatial analyses using secondary data in the public domain. The information was collected from January to March 2023 and encompassed all municipalities in Brazil. The country has a population of 215,232,681 inhabitants which are distributed across 5,570 municipalities and organized into five major regions that are subdivided into 26 states and one Federal District^12^ ^.^

The data for this study comes from the Notifiable Diseases Information System (SINAN), collected from the electronic portal of the SUS Information Technology Department (DATASUS). These comprised TB cases reported between 2007 and 2021 and were associated with two or more chronic health conditions (multimorbidity).

Four conditions considered fundamental to the management of TB associated with multimorbidity were selected: HIV/AIDS, diabetes, mental illness, and COPD^11^. The first three conditions have their own fields on the TB notification form because of their epidemiological relevance in the Brazilian context^5^, and the corresponding variables were used in this study. COPD, on the other hand, was identified through a nominal search in the "other associated diseases" field, since this condition leads to an obvious exacerbation of TB symptoms, longer duration, and greater severity of cases^13^.

For the temporal analysis, the annual incidence of the disease was calculated using the number of cases reported in each year of this study divided by the estimated population of the periods collected, which are available on the website of the Brazilian Institute of Geography and Statistics^12^, multiplied by a factor of 100,000. The software calculates the temporal trend through regression using inflection points, indicating whether there are one or more line segments that indicate any change in the temporal trend of the object analyzed^14^.

The results of the temporal analysis were used to estimate the annual percentage change (APC), its 95% confidence interval (95%CI), and statistical significance. A significantly positive APC indicates an upward trend in the phenomenon studied. A negative APC indicates a decreasing trend. Nonsignificant APC values indicated a stationary trend^14^.

For spatial analysis, an average municipal prevalence rate variable was created for each municipality in Brazil. This was standardized by the indirect method using the middle year of the period (2014) as a reference. These crude rates are displayed on a thematic map. However, owing to the heterogeneity of the rates and the instability of values between neighboring municipalities, they were smoothed using the local empirical Bayesian method. The application of this method is necessary as it generates rates that are closer to reality, as it considers not only the rate value of a given municipality but also weights it in relation to those that border it through a spatial proximity matrix^14^. The local empirical Bayesian rate analysis was carried out in the GeoDa 1.14® software.

Spatial clusters were identified using a spatial autocorrelation function, in which the Moran Global and Local index (LISA) was applied to verify the presence of spatial clusters and quantify the degree of spatial association in each municipality. Therefore, it is possible to identify primary clusters, that is, those with the lowest probability of having occurred at random. This association is shown in the Moran scatter diagram, which identifies four quadrants: quadrant one (Q1) shows municipalities with high rates that are close to municipalities with equally high rates (High/High spatial pattern); quadrant two (Q2) shows municipalities with low rates that are surrounded by municipalities that also have low rates (Low/Low spatial pattern); in quadrant three (Q3) are municipalities with high rates that are surrounded by municipalities with low rates (High/Low spatial pattern); finally, in quadrant four (Q4) are municipalities with low rates that are surrounded by municipalities with high rates (Low/High spatial pattern), considering p<0.05^14^.

Subsequently, a spatiotemporal scan analysis was performed to identify areas with a higher risk of TB prevalence associated with multimorbidity. The discrete Poisson model was adopted to identify the clusters, considering the following requirements: a maximum cluster size of 50% of the exposed population, circular clusters, and 999 replications. The relative risk (RR) was calculated for each municipality in Brazil; those with values >1 had a higher risk than the entire country^15^.

Finally, regression models were used to identify the factors that influenced the prevalence of TB associated with multimorbidity. Initially, socioeconomic indicators were entered into a generalized linear model (GLM) with gamma family and log links, as this type of regression is an extension of classic linear methods. GLMs’ estimation is performed using the maximum likelihood method, creating a series of distributions for the response variable that dismisses the assumptions of normality, linearity, and homoscedasticity for data analysis^16^ ^,^ ^17^. Multicollinearity was tested using the Variance Inflation Factor (VIF), and variables with VIF>10 were evaluated and excluded from the analysis to accept the statistical results. Furthermore, owing to the spatial nature of the outcome and the aims of the analysis, the indicators that remained in the final multivariate GLM were entered into two global spatial regression models (spatial lag and spatial error).

The indicators that made up the initial multivariate regression model were the illiteracy rate (T_ANALF18M), Gini Index (GINI), Brazilian Index of Deprivation (IBP), THEIL Index (THEIL), Municipal Human Development Index (IDHM), Proportion of Extremely Poor (PIND), FIRJAN Municipal Development Index (IFDM), Coverage Rate of the Family Health Strategy (ESF), Social Vulnerability Index (IVS), *Bolsa Família* Program (PBF), Average Per-person Income (RDPC), Percentage of the population living in households with a density of more than 2 people per bedroom (T_DENS) and Lambda Constant (Spatial Error). Three methods (GLM, Spatial Lag, and Spatial Error) were compared, and the method with the best fit was selected based on the highest value of the adjusted coefficient of determination (R²) and the lowest value of the Akaike Information Criterion (AIC).

Temporal regression by inflection points was carried out using Joinpoint 5.0® software. The calculation of the spatial autocorrelation test and the spatial lag and spatial error regressions were carried out in the GeoDa 1.14® software. The spatiotemporal scan analysis was carried out in the SaTScan 9.7® software, and the classical non-spatial GLM analysis was carried out in the Stata v.13® software. All analyses were conducted using crude rates, and Bayesian rates were used only to aid the visualization of distributions in one map. All maps were produced in QGIS 3.16® software.

As the data are of a secondary nature and in the public domain, they do not need to be approved by the Research Ethics Committee (REC) for analysis, as set out in Resolution 510/16 of the National Health Council; however, the researcher's commitment to the principles of bioethics set out in Resolution 466/12 is also reinforced. In addition, it should be noted that this data is covered by Law No. 13.709/18, known as the General Data Protection Law (LGPD, in Portuguese), which governs the processing of personal data, including in digital media. Additionally, the researcher was supported by the Access to Information Law (12.527/11), which established access to public information as a fundamental principle.

## RESULTS

Between 2007 and 2021, 1,319,752 TB cases were reported, of which 7,795 (0.6%) were associated with multimorbidity. Regarding the sociodemographic characteristics of the population, the highest proportion of cases was reported in men (65.2%; n=5,087) with brown skin color (42.3%; n=3,302), incomplete primary education (39.1%; n=3,045), and a median age of 46 years (IQR: 36-55). The Brazilian region with the highest percentage of notifications was the Southeast (38.2%; n=2981). The Midwest region had the lowest percentage (3.6%; n=286). Table 1 shows that many notifications had two comorbidities simultaneously with TB (94.1%, n=7,340). Mental illness was the most common comorbidity (70.8% n=5,525). COPD was the least common comorbidity (2.6%; n=202).

**TABLE 1:** Sociodemographic and clinical characteristics of the study population.

Sociodemographic and clinical features	n	%
**Gender**		
Male	5,087	65.25
Female	2,706	34.71
Unknown/Not Informed	2	0.04
**Age**		
Mean ±SD	45.8±14.2	
Median (IQR)	46 (36 - 55)	
Min. - Max.	0 - 103	
**Race/color**		
White	2,564	32.9
Black	1,295	16.6
Yellow	44	0.6
Brown	3,302	42.4
Indigenous	30	0.4
Unknown/Not Informed	415	7.1
**Education**		
Illiterate	550	7.1
Incomplete elementary school	3,045	39.1
Complete elementary school	476	6.1
High school incomplete	483	6.2
High school complete	529	6.8
Incomplete higher education	114	1.5
Higher education complete	155	2.0
Unknown/Not Informed	1,800	31.2
**Region**		
North	637	8.2
Northeast	2,187	28.1
Southeast	2,981	38.2
South	1,700	21.8
Midwest	286	3.7
Pop. Special*		
Yes	1,171	4.9
No	10,765	95.1
**COPD**		
No	7,593	97.4
Yes	202	2.6
**HIV/Aids**		
No	2,949	37.8
Yes	4,846	62.2
**Diabetes**		
No	2,323	29.8
Yes	5,472	70.2
**Mental illness**		
No	2,270	29.1
Yes	5,525	70.9
**No. of comorbidities Simultaneous with TB**		
2	7,340	94.2
3	455	5.8

*This includes the population deprived of liberty, homeless people, and beneficiaries of government cash transfer programs.

**FIGURE 1:**
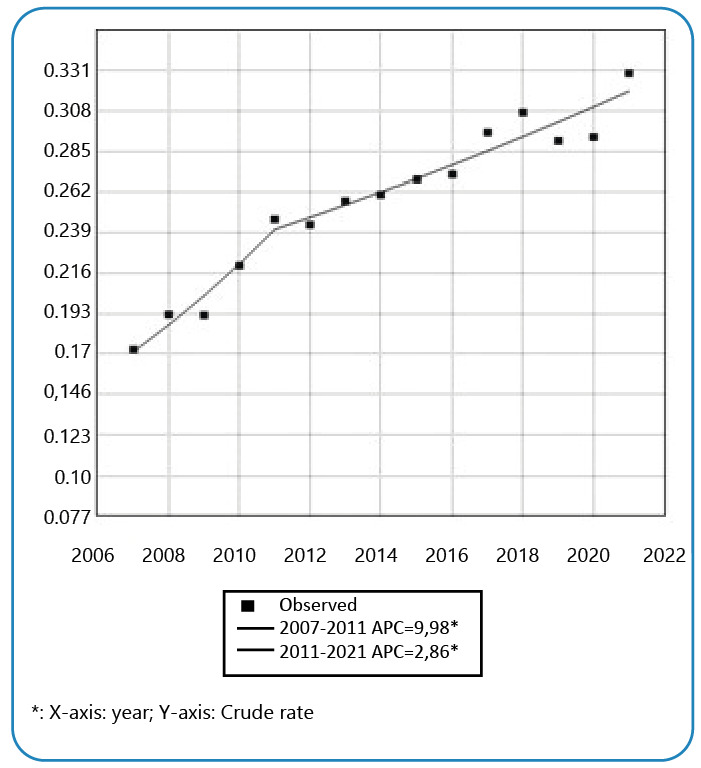
Temporal trend of TB associated with multimorbidity in Brazil, 2007-2021*.

Regarding the evolution over time in Brazil, there was a significant increase in notifications, which were separated into two segments. The first segment occurred from 2007 to 2011, with a significant growth of 8.9% per year (95%CI: 4.2-13.9; p=0.002). The second period observed, 2011 to 2021, also showed a significant growth trend, with an APC of 2.9% (95%CI: 2.0-3.8; p<0.001). Considering the entire period studied, an average growth trend of 4.6% per year was identified (95%CI: 3.3-5.9; p<0.001). Figure 1 shows the temporal evolution during this period.

**FIGURE 2:**
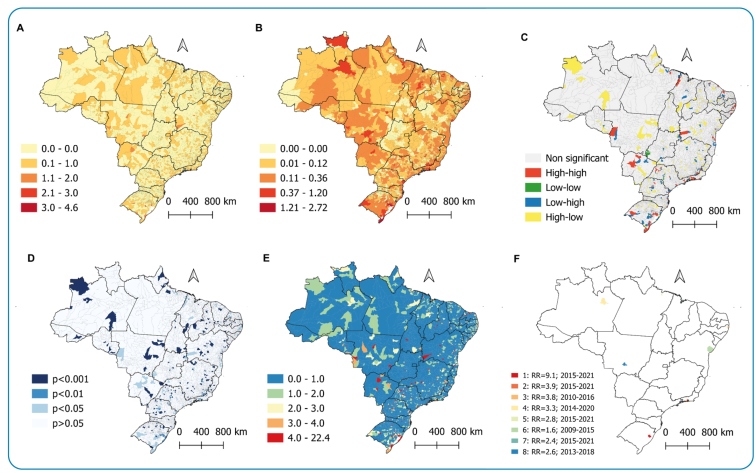
Spatial distribution of the crude prevalence rate **(A)** and after smoothing by the local empirical Bayesian method **(B)**; spatial clusters of prevalence by Moran map **(C)** and Lisa map **(D)**; relative risk characterized by scan statistics (E) spatiotempal clusters of TB prevalence associated with multimorbidity defined by scan statistics, 2007-2021.

We found that 21.1% (n=1,178) of Brazilian municipalities reported that TB was associated with multimorbidity (Figure 2A). However, some municipalities stood out in terms of the prevalence of this condition: Recife (15.9%; n=239), São Paulo (19.8%; n=297), Porto Alegre (23.6%; n=354) and, above all, Rio de Janeiro (44.8%; n=672), which stood out in comparison to the total number of cases notified throughout the Brazilian territory.

The application of the local empirical Bayesian method made it possible to smooth the prevalence rates (Figure 2B). This shows a more homogeneous spatial distribution throughout the country, highlighting regions with higher concentrations of TB and multimorbidity located in the states of Amazonas (north), Rio Grande do Sul (south), and Rio de Janeiro (southeast).

To detect spatial clusters, the Local Moran's index identified high-high distribution patterns, which were found mainly in Rio de Janeiro (southeast) and Rio Grande do Sul (south) (Figure 2C). The LISA map (Figure 2D) shows the statistical significance of each identified cluster. Finally, the scan technique made it possible to identify municipalities in the states of Paraíba and Pernambuco (northeast), São Paulo and Minas Gerais (southeast), Paraná and Rio Grande do Sul (south), and Mato Grosso (midwest), which presented the highest risks in terms of TB and multimorbidity (Figure 2E). The primary spatiotemporal cluster was identified in Rio Grande do Sul, whereas the others were in Rio de Janeiro, Pernambuco, Amazonas, and Pará. No significant spatiotemporal clusters were found in Bahia, Maranhão, and Mato Grosso (Figure 2F).

Regarding multivariate analysis, the indicators that made up the final model were the Illiteracy Rate (ANALF18M), Gini Index (GINI), Proportion of Extremely Poor (PIND), FIRJAN Municipal Development Index (IFDM), and Family Health Strategy Coverage Rate (ESF). Social Vulnerability Index (IVS), *Bolsa Família* Program (PBF), average per-person income (RDPC), percentage of the population living in households with a density of more than two people per bedroom (T_DENS), and Lambda Constant (Spatial Error). The following were excluded: the Brazilian Index of Deprivation (IBP), THEIL Index (THEIL), and Municipal Human Development Index (IDHM), as they had a Variance Inflation Factor (VIF) > 10.

The table below shows the results of the three regression models. The global spatial models show that the proportion of extremely poor (β=-0.002; p=0.002), the *bolsa família* program (β=0.002; p<0.001), the average per-person income (β=0.001; p<0.001), and the percentage of the population living in households with a density of more than 2 people per bedroom (β=0.001; p=0.002) were related to the outcome (Table 2).

**TABLE 2:** GLM, Spatial Lag, and Spatial Error regression models of the indicators that influence the prevalence of TB associated with multimorbidity in Brazil, 2007 to 2021.

Indicator	GLM			Spatial Lag			Spatial Error		
	Coef.	Erro padrão	p-valor	Coef.	Erro padrão	p-valor	Coef.	Erro padrão	p-valor
Illiteracy Rate	0.002	0.01	0.862	0.0002	0.001	0.811	5*10^-5^	0.001	0.946
Gini Index	2.02	1.15	0.08	0.13	0.08	0.102	0.13	0.08	0.126
Proportion of Extremely Poor	-0.04	0.01	<0.001	-0.002	0.001	<0.001	-0.002	0.007	0.002
FIRJAN Municipal Development Index	0.69	0.49	0.160	0.05	0.04	0.190	0.05	0.03	0.201
Family Health Strategy Coverage Rate	-0.26	0.23	0.261	-0.02	0.02	0.261	-0.02	0.02	0.175
Social Vulnerability Index	-0.51	0.98	0.605	0.01	0.08	0.873	-0.0004	0.07	0.995
*Bolsa Família* Program	0.02	0.004	<0.001	0.002	0.0003	<0.001	0.002	0.0003	<0.001
Average per-person income	0.001	0.0001	0.197	0.0001	0.00005	<0.001	0.0001	3*10^-5^	<0.001
Percentage of the population living in households with a density of more than 2 people per bedroom	0.02	0.007	0.008	0.001	0.0004	0.004	0.001	0.0005	0.002
Constant	-5.28	0.72	<0.001	-0.19	0.05	<0.001	-0.18	0.05	<0.001
Spatial lag				0.13	0.02	<0.001			
Spatial error							0.14	0.02	<0.001

*: potentiation.

## DISCUSSION

This study identified an upward trend in the prevalence of TB associated with multimorbidity in Brazil, which was more abrupt until 2011 and stabilized thereafter. We believe that in the first segment, surveillance of comorbidities related to TB infection was in the process of implementation and an increased number of cases were identified. In the second segment, surveillance may be better implemented and, although there was a significant increase, it was not as high as in the first period. This trend can also be observed in other studies, varying according to the type of comorbidity^18^ ^,^ ^19^, including studies that evaluated the incidence of TB and a state with a high incidence of the disease, highlighting the annual reduction in notifications from that year onwards. However, there was no significant evidence to explain this phenomenon^20^.

Regarding chronic diseases, the literature shows that, in Brazil, there was an increase in mortality due to comorbidities such as diabetes mellitus during this period, especially in the northeast region. Recently, Feliciano et al^21^, described that there is also an association between an increase in chronic diseases and an increase in social disparities, a situation that can be observed, for example, in the southern and southeastern regions, where the increase in TB incidence is related to social indicators such as the local Human Development Index (HDI).

Regarding spatial analysis, both the crude prevalence rate and the analysis after smoothing using the Bayesian method indicated the relevant regions for TB associated with multimorbidity in the territory studied. These regions are mainly composed of the states of Rio de Janeiro, Amazonas, and Rio Grande do Sul, territories that, until 2023, had a high incidence of the disease^22^. In addition, states such as Roraíma, Pará, Mato Grosso do Sul, and Pernambuco have historically been relevant to TB notifications in the country^4^ ^,^ ^5^. 

In addition, the presence of primary clusters is observed in Rio de Janeiro and Rio Grande do Sul. This, in addition to having a high incidence of TB, is relevant in terms of the rates of abandonment of the disease treatment, which fosters the maintenance of the transmission chain and enables the expansion of drug resistance cases registered in the country^23^ ^,^ ^24^ ^,^ ^25^.

The spatial clusters found in this study were similar to those found in previous studies^26^ ^,^ ^27^. This phenomenon is associated with the sociodemographic profile of the resident population, which involves aspects such as low schooling and exposure to poverty, hindering access to rapid diagnostic and adequate treatment services for the disease, as well as the occurrence of multimorbidity^28^. However, clusters were also found in regions with a higher HDI, especially in the south, which may indicate a possible influence of the quality of disease surveillance systems in this region, enabling greater efficiency in screening and diagnosis as well as an increase in the burden of chronic diseases in this territory.

It can be seen, then, that the spatiotemporal evolution is intrinsically related to socioeconomic factors, since in this study, there was a positive association with some indicators such as the *Bolsa Família Program*, the average income per person, and the percentage of the population living in households with a density of more than two people per bedroom, which can also be observed in the literature^27^ ^,^ ^29^. However, there is a favorable relationship with regard to the income program (Bolsa Família), in which there is evidence of greater cure and less abandonment of TB treatment in this population^30^.

Finally, we highlight the relationship between these findings and the syndemic aspects of TB and other conditions. DM has a strong relationship with TB and is one of the main obstacles to its global coping^31^ ^,^ ^32^. Chronic diseases are mainly associated with advanced age, making individuals more susceptible to other pathologies. Individuals with DM are three times more likely to contract active TB^33^. In addition, there is an increase in drug resistance to TB (MDR-TB) in this population, which delays the diagnosis of active TB, affects treatment and increases morbidity and mortality^34^. 

There is also a strong relationship between TB-HIV/AIDS cases and socioeconomic aspects related to social vulnerability in the population most affected by this coinfection. Populations considered key or priority, such as individuals deprived of liberty and homeless people, have a higher rate of unfavorable outcomes, including death^26^ ^,^ ^35^ ^,^ ^36^.

COPD is a comorbidity that has recently been attributed to TB because the damage caused by the bacillus to the lung parenchyma favors its future involvement in chronic diseases, especially when associated with smoking. In this context, there is consensus that TB is a risk factor for COPD^37^ ^,^ ^38^.

Finally, regarding the investigation of mental illness as a comorbidity, there is evidence in the literature of an intrinsic relationship between TB and depression, which is observed through social, behavioral, and biological mechanisms. It is estimated that depression may affect up to half of the individuals affected by TB, in view of the social burden that is manifested by this illness. This condition is associated with a greater burden on individuals, culminating in higher rates of morbidity and mortality, treatment abandonment, and drug resistance^39^ ^,^ ^40^.

In Brazil, primary healthcare (PHC) has been successfully used to treat this comorbidity in patients with TB. Even though there is no specific population sample, the development of mental disorders can be aggravated when using alcohol and illicit substances. Similar to TB, the association between the use of such substances and the development of these disorders is more common among socially vulnerable individuals, and this population is mostly covered by PHC^26^ ^,^ ^41^.

Finally, there is the influence of sociodemographic risk factors, such as monthly income, housing conditions, and family size, which materialize in the restriction of access to health services and, consequently, contribute to the transmission of the bacillus^34^. In this context, the literature highlights that the incidence is higher in states with greater social vulnerability^28^. 

This study has some limitations, mainly owing to the use of secondary databases, which are susceptible to incompleteness and nonadherence, thus being susceptible to an ecological fallacy. The study also used population data that may be outdated; therefore, the results found may not correspond faithfully to reality, being only estimates. It is noteworthy that the use of Bayesian rates may cause spatial dependence in the data because the characteristics of the method depend on maximum likelihood. 

The authors propose that more studies be carried out on the subject, given that there is currently little national literature using this methodology, especially when it comes to analyzing multimorbidity and not just one associated condition.

## CONCLUSIONS

Analysis of the spatiotemporal pattern allowed the identification of priority states and indicators that influenced prevalence rates. These findings will stimulate public action to manage this situation. However, because it is still a recent topic in the literature, the authors encourage the development of studies on the synergistic characteristics between TB and other clinical conditions, principally in Brazil, which shows an increasing trend not only in relation to TB but also to chronic diseases.
